# Allogenic haematopoietic stem cell transplantation in VEXAS: A review of 33 patients

**DOI:** 10.1007/s10067-024-07160-7

**Published:** 2024-09-30

**Authors:** Syed B. Ali, Carmelo Gurnari

**Affiliations:** 1 Department of Clinical Immunology and Allergy, Flinders Medical Centre, Bedford Park, South Australia Australia; 2 School of Medicine and Public Health, Flinders University, Bedford Park, South Australia Australia; 3 Department of Clinical Immunology and Allergy, Royal Adelaide Hospital, Adelaide, South Australia Australia; 4 School of Medicine and Biomedical Sciences, University of Adelaide, Adelaide, South Australia Australia; 5 Department of Translational Hematology and Oncology Research, Taussig Cancer Institute, Cleveland Clinic, Cleveland, OH USA; 6 Department of Biomedicine and Prevention, University of Rome Tor Vergata, Rome, Italy

**Keywords:** Allogenic stem cell transplantation, Myelodysplatic syndrome, Somatic mutation, Transplantation, VEXAS Syndrome

## Abstract

Vacuolation, E1 enzyme, X-linked, autoinflammatory, somatic (VEXAS) syndrome is a multisystem disease due to a genetic mutation in the ubiquitin-activating enzyme (*UBA1*). Allogeneic haematopoietic stem cell transplantation (allo-HSCT) offers both therapeutic and cure but also carries significant risks. A review of VEXAS and HSCT cases was undertaken. Thirty-three patients were identified; majority males (*n* = 32, 97.0%), median time from symptoms to HSCT: 3 years (IQR 2.0–4.8) and median age of 59 years (IQR 52.5–65.5). *UBA1* mutation Met41Thr was most common (11/32, 34.4%). The median variant allele frequency was 56.5% (IQR 43.0–73.5) with no correlation with increasing age. Prior to HSCT, 4.5 (IQR 2.8–6) treatments were trialled. Peripheral blood HSCT (30/31, 96.8%) and HLA-matched, unrelated donor (18/32, 56.3%) were most common. Conditioning regimens varied, with reduced intensity treatment with fludarabine as a co-agent most frequently administered (12/31, 38.7%). Both acute and/or chronic GVHD (18/32, 56.3%) and infections were common (12/32, 37.5%). Overall, 27 individuals (81.8%) were alive, and those undergoing HSCT prospectively had median follow up of 9 months (IQR 3.8–14.4). Of the six deceased, infection was implicated in four. In 11 cases with post-HSCT molecular data, a complete eradication of *UBA1* mutation was reported. In summary, while consensus treatment strategy regarding VEXAS is lacking, this review highlights HSCT may remain not only a therapeutic option but also enable cure. However, considerations regarding comorbidities, concurrent haematological disorders as well as overall risks of GVHD and infections need to be made.

**Table:** 

**Key points** *• Very few reported prospective cases of VEXAS and allogeneic haematopoietic stem cell transplantation (allo-HSCT) have been reported.* *• While risks of graft versus host disease and infection remain barriers, this treatment modality remains an option for selected patients.* *• Allo-HSCT is the only treatment strategy which can remove the UBA1 mutation.*

**Key points**

*• Very few reported prospective cases of VEXAS and allogeneic haematopoietic stem cell transplantation (allo-HSCT) have been reported.*

*• While risks of graft versus host disease and infection remain barriers, this treatment modality remains an option for selected patients.*

*• Allo-HSCT is the only treatment strategy which can remove the UBA1 mutation.*

## Introduction

Vacuolation, E1 enzyme, X-linked, autoinflammatory, somatic (VEXAS) syndrome is a multisystem disease arising due to a genetic mutation in the ubiquitin-activating enzyme (*UBA1*) on the X-chromosome [1]. Several pathways including protein homeostasis and cell signalling are affected with dysregulation of ubiquitination processes and activation of inflammation [2, 3].

Somatic mutations in the *UBA1* gene have a reported prevalence of 0.007% [4]. While canonical phenotypes of male gender and age above 60 years are well described, there are increasing reports of the disease affecting females or a younger age of presentation [5]. As concurrent haematological disease such as myelodysplastic syndrome (MDS) may occur with VEXAS, the presence of a somatic *UBA1* mutation is now being detected in patients with longstanding diagnoses of MDS pre-existing the discovery of VEXAS [6].

Treatment paradigms for VEXAS focus on targeting pro-inflammatory pathways, but given the relatively recent discovery of the disease and its heterogeneous manifestations, clear guidelines are lacking. Glucocorticoids form the foundation of treatment and additional treatments such as conventional disease-modifying antirheumatic drugs (csDMARDs) are frequently used, although most have demonstrated poor efficacy [7]. Biologic therapies targeting the IL1 and IL6 cytokines have also been used but with variable efficacy [7]. Recently, oral Janus kinase inhibitors have gained popularity due to simultaneous targeting of multiple cytokine targets; however, a systematic review reported complete remission in only 33% and partial remission in 27.3% from this treatment [7]. Concurrent haematological diseases, such as MDS is often addressed with azacitidine, a hypomethylating targeted treatment which is has a direct cytotoxic effect [8].

More importantly, given low response rates with treatment strategies which do not eliminate the *UBA1* mutation, patients may have transfusion dependency for symptomatic anaemia. Due to the somatic nature of the mutation, variant allele frequency may also increase over time, possibly contributing to progressive disease. Furthermore, as the 5-year survival rate is as low as 63% [9], treatment strategies need to address improving both mortality and morbidity. This is particularly the case as patient population is older, with more comorbidities, making immunosuppressant therapies more problematic in terms of adverse effects as well as interactions.

Allogeneic haematopoietic stem cell transplantation (allo-HSCT) is a possible therapeutic option with the attractive notion of replacing progenitor cells affected by the *UBA1* somatic mutation, thereby enabling disease remission [10]. However, as allo-HSCT carries both high rates of morbidity and mortality, upfront treatment might not always be desirable. There is a risk with delaying allo-HSCT; however, in that with accumulating inflammatory burden and immunosuppression, patients may become too frail to be considered for allo-HSCT.

Given the everchanging landscape of VEXAS and notable difficulties in achieving controlled remission with treatments thus far, a narrative review of allo-HSCT in this disease entity was performed. The purpose of this review is to provide clinicians with current data, raising awareness about the risks and benefits of allo-HSCT. This is of particular importance as clear guidelines on transplantation in the setting of VEXAS are lacking.

## Methods

A review of the literature was completed with search terms “VEXAS” and “transplantation” using PubMed and Embase online databases in April 2024. All publications were reviewed for the search period January 2020 to April 2024. Only those with peer-reviewed publications and full text availability were included. Duplicate publications, including cases already reported, as well as abstracts from meetings were excluded.

Detailed data collection including demographics, *UBA1* somatic mutation, variant allele frequency, clinical presentation, delay of diagnosis from initial symptoms, treatment modalities leading up to transplantation, as well as transplantation regimen and outcomes were recorded. Corresponding authors were directly contacted to obtain missing data and incorporated only if received, otherwise left blank.

Case series and reports were further sub-grouped for purposes of analysis as follows: transplantation following diagnosis of VEXAS, i.e., prospectively (group 1), or patients who had previously undergone transplantation for other haematological diseases such as MDS in whom the diagnosis of VEXAS was made retrospectively (group 2).

## Results

A total of 12 publications were identified globally: France (*n* = 3), USA (*n* = 2), Canada (*n* = 1), Czech Republic (*n* = 1), Italy (*n* = 1), Netherlands (*n* = 1), Spain (*n* = 1), Sweden (*n* = 1), and UK (*n* = 1). These publications comprised a total of 33 individuals: 23 patients in group 1, and 10 patients in group 2.

Clinical details are summarised in Table 1. The median age at transplantation was 59.0 years (IQR 52.5–65.5). There were no significant differences between the ages of patients in the two groups (58.6 vs. 59.5 years, *p* = 0.79). Most individuals were males (*n* = 32, 97.0%).

**Table 1:** Summary of reported cases of VEXAS and transplantation to date for those performed prospectively (group 1), and those whereby diagnosis of VEXAS was made retrospectively (group 2)

Reference	Age, sex	Clinical presentation/disease phenotype	Time between symptoms and transplant (years)	Mutation, variant allele frequency (%)	Concurrent haem disease	Lines, and types of treatment pre-transplant	Transplantation			GvHD prophylaxis	Post-transplantation	
							Indication	Type, donor	Conditioning		Complications	Follow-up (months) alive /dead
VEXAS and prospective transplantation (group 1)												
Stiburkova et al. July 2023, Czech Republic[31]	61 M	Fever, rash, arthritis, lymphadenopathy	2	c.1420G > C;pGly477Ala, N/A	MDS	5; CS, MTX, Infx Tofa, Tcz	MDS and VEXAS progression: transfusion support, inflammatory	PB, MUD	S–flu, treo	ATG, Cyc, MMF	Nil	8, alive
Gurnari et al. December 2023, Italy[14]	69 M	Fever, rash, arthritis, lung, VTE	3.8	c.122 T > C; pMet41Thr, 50%	MDS	13; csDMARD, bDMARD, tsDMARD, Azac	N/A	PB, MUD	R–flu, bu	ATG, Cyc, MTX	Nil	18.6, alive
	53 M	Arthritis, VTE	0.75	c.118-2A > C, N/A	MDS	6; csDMARD, bDMARD, Azac	N/A	PB, MUD	S–flu, bu	ATG, Cyc, MTX	Grade I aGVHD	1.1, alive
	52 M	Fever, arthritis, chondritis, rash, VTE, lung	4	c.122 T > C; pMet41Thr, 43%	Nil	5; csDMARD, bDMARD	N/A	PB, MMUD	S–flu, mel, TT	ATG, MMF, TCRab/CD19 depletion	Nil	18.3, alive
	52 M	Fever, rash, arthritis	1.9	c.121A > C; pMet41Leu, N/A	Nil	2, csDMARD	N/A	PB, MUD	R–flu, bu	ATG, CNI, MMF	Grade IV aGVHD, Limited cGVHD	10.9, alive
	67 M	Rash	3	c.121A > G; pMet41Val, N/A	Nil	N/A	N/A	PB, MUD	R–flu, mel	Alem, Cyc	Limited cGVHD	12.2, dead
	65 M	Fever, rash, chondritis, lung, VTE	4.67	c.122 T > C; pMet41Thr, 73%	MDS	2; csDMARD	N/A	PB, MUD	R–flu, treo	ATG, Cyc. MTX	Grade I aGVHD	14.4, alive
	59 M	N/A	4.3	c.167C > T; pSer56Phe, 90%	MDS	2; Azac	N/A	PB, MRD	R–flu, bu, cy, amsa, arac	ATG, CNI, MMF	Grade II aGVHD	29.1, alive
	59 M	Arthritis, chondritis	5.1	c.118-1G > C; N/A	MDS	5; csDMARD, bDMARD, tsDMARD, Azac	N/A	PB, MMRD	R–flu, bu, TT	Cy, CNI, MMF	Grade II aGVHD	4.3, alive
	59 M	Fever, rash, arthritis, VTE	4.75	c.122 T > C; pMet41Thr, 75%	MDS	1; tsDMARD	N/A	PB, MUD	S–flu, treo	ATG, Cyc, MTX	Nil	0.9, alive
Guerineau et al. April 2024, France[32]	29 M	Fever, rash, myositis, epididymitis	1	c.121A > G; pMet41Val, 63%	MDS	3; CS, Azac Venetoclax	VEXAS progression: inflammatory	PB, MRD	Bu, cy	ATG, Cyc, MTX	Grade III aGVHD, cGVHD	24, alive
Diarra et al. February 2022, France[33]	50 M	Rash, chondritis, lung, DVT	2	c.122 T > C; pMet41Thr, 67%	MDS	6; CS, dapsone, colchicine, ana canakinumab, siltuximab	VEXAS progression: inflammatory	PB, MUD	S–flu, bu,	ATG, Cyc, MTX	*Infections:* bacterial catheter	3, alive
Loschi et al. November 2021, France[34]	70 M	Rash, arthritis	9	c.121A > C; pMet41Leu, N/A	MDS	8; CS, HCQ, thalidomide, MTX, infx, ana, ustekinumab, IVIG, bara, cy, ruxo	Likely MDS progression as Increased transfusion support and inflammatory symptoms controlled	PB, MMUD	R–flu, bu	Cy, Cyc, MMF	Grades I and II aGVHD (gastrointestinal and cutaneous respectively)	4, alive
van Leeuwen-Kerkhoff et al. August 2022, Netherlands[35]	50 M	Fever, rash, chondritis, arthritis	3	c.122 T > C; pMet41Thr, 43%	Nil	5; CS, aza, RTX, Canakinumab	VEXAS progression: steroid dependency	PB, MMUD	Flu, mel, thio	ATG, MMF, Canakinumab	Grade 1 aGVHD *Infections*: CMV	9, alive
Al-Hakim et al. September 2022, UK[29]	51 M	Fever, Rash, Lung	2	c.121A > G; pMet41Val, 50%	MDS	4;CS, ana, bara colchicine	VEXAS progression: inflammatory symptoms and transfusion dependency	PB, MRD	Flu, bu, thio	Cyc, Tac, MMF	*Infection:* Salmonella, pseudomonas	0.4, death due to infection
	67 M	Fever, rash, PE	3	c.121A > G; pMet41Val, 24%	Nil	3; CS, MTX, HCQ	VEXAS progression: inflammatory symptoms	PB, MUD	Flu, mel	Alem, Cyc	aGVHD, HLH *Infection:* EBV	5, alive
	61 M	Fever, rash, lung	9	c.122 T > C; pMet41Thr, 50%	Nil	6; CS, MTX, aza, MMF, ana, tcz, bara	VEXAS progression: inflammatory symptoms and transfusion dependency	PB, MRD	Flu, treo	Cyc, MMF	Myelitis, optic neuropathy *Infection:* Clostridium difficile	11, death due to sepsis, multiorgan failure and cardiac arrest
Mangaonkar et al. November 2022, USA[15]	63 M	Relapsing polychondritis	N/A	c.122 T > C; pMet41Thr, N/A	MDS	11, CS, MTX, MMF, decitabine, HCQ, testosterone, cyc, RTX, tcz, adalimumab, abatacept, lenalidomide	MDS and VEXAS progression: transfusion dependency and inflammatory symptoms	PB, MUD	Flu, mel	Cy, Tac, MMF	Mucositis *Infection*: bacteraemia (organism N/A)	16.2, alive
	60 M	Urticarial vasculitis	N/A	c.121A > G; pMet41Val, N/A	Nil	3; CS, dapsone, Oma, MMF	VEXAS progression: inflammatory symptoms	PB, MRD	Flu, mel	Cy, Tac, MMF	Nil	12.8, alive
	59 M	Arthritis	N/A	c.118-1G > C (splice variant); N/A	Nil	7; CS, MTX, leflunomide, adalimumab, etanercept, infx, tcz	VEXAS progression: inflammatory symptoms	PB, MRD	Flu, mel	Tac, MTX	Drug induced rash	3.8, alive
	74 M	N/A	N/A	c.122 T > C; pMet41Thr, N/A	Nil	3; CS, ruxo, tcz	VEXAS progression: inflammatory symptoms	PB, MUD	Flu, mel	Cy, tac, MMF	Grade 1 aGVHD Mucositis, *Infection*: Clostridium difficile, Escherichia coli	2.9, alive
	49 M	Relapsing polychondritis	N/A	c.122 T > C; pMet41Thr, N/A	MDS	1; CS	MDS and VEXAS progression: inflammatory symptoms	PB, MUD	Flu, mel	Cy, tac, MMF	Mild dermal hypersensitivity reaction	9.6, alive
Obiorah et al. August 2021, USA[36]	69 M	N/A	N/A	c.121A > C; pMet41Leu, 86%	MM	3; CS, lenalidomide, bortezomib	MM–incomplete response	N/A	N/A	N/A	MM relapse	6, alive
Transplantation and retrospective diagnosis of VEXAS (group 2)												
Al-Hakim et al. September 2022, UK[29]	62 M	Fever, rash, relapsing chondritis	3	c.121A > C; pMet41Leu, 40%	MDS	1; CS	Likely both VEXAS and MDS: Transfusion dependency	PB, MUD	Flu, bu	ATG, cyc	Grade I cGVHD	40, alive
Diarra et al. February 2022, France[33]	46 M	Fever, rash, vasculitis, orchitis	3	c.121A > G; pMet41Val, 73%	MDS	6; CS, ana, dapsone, canakinumab, aza, HCQ	VEXAS progression: inflammatory—vasculitis	PB, MUD	S–flu, bu	ATG, cyc, MTX	cGVHD	32, alive
	59 M	Rash	3	c.121A > G; pMet41Val, 88%	MDS, Myelofibrosis	8; CS, CP, IVIG, RTX, danazol, ana, dapsone, canakinumab	VEXAS progression: inflammatory – vasculitis	BM, MRD	R–flu, bu	Cyc, MTX	cGVHD	67, alive
	65 M	Rash, vasculitis, chondritis	2	c.121A > G; pMet41Leu, 43%	MDS	7; CS, MTX, ana, canakinumab, tcz, IVIG, aza	VEXAS progression	PB, MUD	R–flu, bu	Cy, MMF, cyc	Grade I aGVHD *Infections*: BK and CMV	38, alive
	58 M	Chondritis, lung	2	c.121A > G; pMet41Val, N/A	MDS	5;CS, tcz, adalimumab, aza, ruxo	VEXAS progression	PB, MRD	R–flu, bu, thio	Cy, MMF, cyc	Grades I and II aGVHD (cutaneous and gastrointestinal respectively) *Infections*: bacterial catheter	5, alive
	55 M	Chondritis	5	c.121A > G; pMet41Val, N/A	MDS, Myelofibrosis	4; CS, MMF, colchicine, aza	VEXAS progression	PB, MUD	Bu, CPA, ATG	Cyc, MTX	Grade III a GVHD *Infections*: bacterial catheter, fusarium	4, death due to infection
Cherniawsky et al. December 2022, Canada[37]	68F	Arthritis, relapsing polychondritis	5	N/A	MDS	5; CS, MTX, aza, etanercept, cyc	MDS progression: transfusion dependency	PB, MRD	R–bu, flu	Cy, MMF, tac	*Infection:* varicella	13, alive
	61 M	Fever, rash, lung, relapsing polychondritis, DVT	11	c.122 T > C; pMet41Thr, N/A	Nil	8; CS, Aza, MTX, tcz, Infx, ana, RTX, bara	Likely VEXAS progression: inflammatory symptoms	PB, MRD	R–bu, flu	Cy, MMF, tac	*Infection:* fungal (invasive)	1, death due to infection
Mascaro et al. August 2023, Spain[38]	55 M	Fever, rash	3	c.118-1G > C; 72%	N/A	1; CS	MDS and VEXAS progression	PB, MUD	Flu, bu	Cyc, MMF	‘Mild to Mod’ GVHD	84, death due infection after bilateral lower limb amputation secondary to acute arterial ischaemia
Gunnarsson et al. July 2023, Sweden[39]	66 M	Portal vein thrombosis	2	c.121A > C; pMet41Leu, N/A	MDS, MM	1; Azac	MM—incomplete response	N/A, MUD	N/A	N/A	N/A	12, alive

*aGVHD* Acute graft versus host disease, *alem* alemtuzumab, *amsa* amsacrine, *Ana* anakinra, *ATG* antithymocyte globulin, *aza* azathioprine, *azac* azacitadine, *bara* baricitinib, *bm* bone marrow, *bu* busulfan, *cGVHD* chronic graft versus host disease, *CMV* cytomegalovirus, *CNI* calcineurin inhibitor, *CS* corticosteroids, *bDMARDS* biologic disease-modifying antirheumatic drugs, *csDMARDS* conventional synthetic disease-modifying antirheumatic drugs, *tsDMARDS* targeted synthetic disease-modifying antirheumatic drugs, *cy* cyclophosphamide, *cyc* ciclosporine, *DVT* deep venous thromobosis, *EBV* Epstein Barr virus, *flu* fludarabine, *HCQ* hydroxychloroquine, *HLH* haemophagocytic lymphohistiocytosis, *IVIG* intravenous immunoglobulin, *oma* omalizumab, *Infx* infliximab, *mel* melphalan, *MDS* myelodysplastic syndrome, *MM* multiple myeloma, *MMF* mycophenolate, *MTX* methotrexate, *MMUD* mismatched unrelated donor, *MRD* matched related donor, *MUD* matched unrelated donor, *N/A* not available, *NSAID* non-steroid anti-inflammatory drug, *PB* peripheral blood, *R* reduced intensity, *RTX* rituximab, *ruxo* ruxolitinib, *S* standard intensity, *Tac* tacrolimus, *Tcz* tocilizumab, *tofa* tofacitinib, *Thio* thiotepa, *Treo* treosulphan, *Tx* transplant

The median time between symptoms and transplantation was 3.0 years (IQR 2.0–4.8) with no significant difference between the two groups (both at 3.0 years, *p* = 0.87).

Somatic *UBA1* mutation data was available for 32 patients. Of these, Met41Thr was most common (*n* = 11, 34.4%), followed by Met41Val (*n* = 9, 28.2%) and Met41Leu (*n* = 6, 18.8%) respectively. Eighteen cases had variant allele frequency reported; median of 56.5% (IQR 43.0–73.5), and no significant relationship with increasing age was observed (*p* = 0.88).

For group 1, VEXAS was the sole diagnosis in nine (39.1%), while the remainder had additional haematological disorders: MDS (*n* = 13, 56.5%) and multiple myeloma (*n* = 1, 4.3%). Data regarding treatment for transplantation was available for 22/23 individuals, and in these patients, several lines of treatment were implemented (median 4.5, IQR 2.8–6). Of these corticosteroids and csDMARDs were common, both used in 14 cases (63.6%). For this prospective transplantation, 14/23 had data available. Of these, VEXAS progression with inflammatory symptoms with or without transfusion dependency (*n* = 9, 64.3%) were most common (Table 1).

Detailed clinical information regarding transplantation of all individuals is also summarised in Table 1. For those with data available, stem cell source was chiefly peripheral blood (30/31, 96.8%), and HLA-matched, unrelated donor (18/32, 56.3%) were most common donor types. Conditioning regimens varied, with reduced intensity treatment with fludarabine as a co-agent most frequently administered (*n* = 12/31, 38.7%). Similarly, there was also variation in graft versus host disease (GVHD) prophylaxis, of which ciclosporin was co-administered with other immunosuppressives in just over half of the cohort (*n* = 19/32, 59.4%).

Of the 32 patients with data available on post-transplant complications, more than half had acute and/or chronic GVHD (*n* = 18/32, 56.3%) (Table 1). Complications of infection were reported in 12/32 individuals (37.5%).

Overall, 27 individuals (81.8%) were alive at the time of publication. In group 1, 20/23 (86.9%) were alive with median follow up of 9.0 months (IQR 3.8–14.4). Of the three deceased patients (13.0%), information was available for two with infection reported as the cause. Two of the three had the Met41Val mutation, with the other case with Met41Thr mutation. In group 2, three of ten (30%) were deceased, in which infection were attributed to two, while the other was from solid organ neoplasia (Table 1).

Notably, in 11 cases with post-HSCT molecular data, a complete eradication of *UBA1* mutation was reported following the transplant procedure, thereby establishing its potentiality for complete cure. Furthermore, in some cases, VEXAS phenotype was reported to be controlled even with persistent mixed chimerism [11].

## Discussion

This narrative review provides up-to-date clinical information about the small number of published cases regarding transplantation in VEXAS. The reported cases were either from centres in North American or Europe, which may reflect firstly the limited experience of transplantation in autoinflammatory diseases, and secondly testing availability for the *UBA1* mutation in some regions.

A clear consensus treatment strategy is still lacking for VEXAS, perhaps due to the heterogeneous nature of the disease, with manifestations which can require the involvement of multiple different medical specialties. Glucocorticoids are the major treatment modality; however, often, high doses over a prolonged period are needed, thereby exposing patients to adverse effects, including dependency on unacceptably high doses (i.e., ≥ 30-mg prednisolone 30 mg) in up to 11% of cases [7]. In this review, the average time between symptoms and transplantation was 3.6 years, indicating that patients may often experience waxing and waning symptoms that might contribute to the morbidity associated with this disease. In addition, several other factors to consider in this context include delays in diagnosis, particularly with availability of *UBA1* genetic testing and recognition of vacuoles within bone marrow raising possibility of diagnosis, as well as access to transplantation services, lack of local experience, and familiarity and persistence with traditional paradigms of immunosuppression even if ineffective. Remarkably, most VEXAS diagnoses in the recent era result from *UBA1* testing of patients already followed up for rheumatologic and haematologic disorders even years prior to the actual suspicion, given the very recent discovery of the disease. Data on lag of VEXAS diagnosis in the current era where *UBA1* testing is available and clinical awareness is more accurate are needed to establish whether the pleomorphic nature of the disease rather than its novelty constitute reason for the observed delay in establishing a proper diagnosis.

There is a pressing need for novel approaches to VEXAS treatment, distinct from traditional paradigms of treating inflammatory diseases or managing haematological malignancies. Recognising that VEXAS patients are of an older demographic, frequently with multiple comorbidities, the timing of definitive treatments such as allo-HSCT is of utmost importance. Management strategies need to include regular and objective assessments of disease activity and treatment success or failure, to ensure that delays to appropriate escalation are minimised. With growing experience with allo-HSCT, there may be less of a need to try prolonged glucocorticoids and/or DMARDs prior to considering transplant. Furthermore, it appears that the specific *UBA1* mutation may have prognostic value, with a large cohort of 116 patients showing that Met41Val mutations had poor outcomes with a 5-year survival of 76.7%, in comparison to Met41Thr and Met41Leu at 83% and 100% respectively [12]. Consequently, there is the potential for therapeutic paradigms which are customised to different levels of genetic risk.

Although allo-HSCT remains the only curative treatment option and should be considered early, its non-negligible morbidity and mortality risk must be carefully considered. In this context, the decision is recommended to be undertaken by multidisciplinary and experienced team, which comprehensively assess patients, especially in this disease where age and comorbidities are common [13]. Interestingly, due to the known refractory nature of VEXAS, others have suggested evaluating for a suitable haematopoietic stem cell donor at the time of VEXAS for patients who might be seemed appropriate for transplantation [13]. Collectively, given the limited number of cases concentrated in USA and European centres, lack of prospective transplantation in VEXAS alone, and long-term outcomes, clarity on when, who, and how to transplant is urgently needed to inform a holistic, multidisciplinary approach [14].

Previously, reports have indicated patient selection as follows: age 75 years or less, genetically confirmed to have *UBA1* mutation consistent with VEXAS and meeting at least one of the following: (a) severe, glucocorticoid refractory, recurrent inflammatory symptoms, (b) persistent (≥ 3 months) cytopenias, including the need for packed red blood cell transfusion and/or platelet transfusions, and (c) coexistent myeloid malignancy or clonal abnormalities predictive of myeloid transformation [15]. A prospective phase 2 clinical trial (NCT05027945) has begun, which will hopefully shed further light on the indications for allo-HSCT in VEXAS [16].

Given the disease’s demographics, a comprehensive assessment is required including organ function, infection risk, personal motivation, and social support [13]. The average age of patients included in this review was below 60 years, with the oldest being 74 years old. Hence, the current literature on allo-HSCT may not be directly applicable to older patients. It is also worth noting that in the literature on haematological malignancy, comorbidities, and performance status independently predict patient outcomes [17, 18].

In addition to patient selection, reduced intensity conditioning regimens and advances in GVHD prophylaxis may be helpful in informing the approach to older patients [19]. Currently, most centres have based allo-HSCT induction therapy for VEXAS on protocols for non-malignant conditions [20, 21]. Regarding conditioning regimens in haematological malignancies, a reduced dosing regimen comprising fludarabine and an alkylating agent has shown comparable efficacy to traditional myeloablative conditioning, with notable advantages including better morbidity and toxicity profiles [22, 23]. On the other hand, as VEXAS might be classified as a non-malignant disease, with the allo-HSCT regimens derived from such disease entities, it has been reported that when compared to malignant disease processes, regimens for non-malignant diseases has a threefold higher risk of graft failure compared to malignant diseases, and lower risk with higher conditioning intensity regimens [24]. In group 1, reduced conditioning regimen was performed in just under 40%.

In our meta-analytic data, near 50% of patients received allo-HSCT from an HLA-matched unrelated donor and GVHD, both acute and chronic were reported in many of the cases (*n* = 20, 57.1%). Treosulfan, an alkylating agent, has been shown to improve overall survival rates in older patients with MDS and had a mild toxicity profile [25]. In this review, only four individuals were treated with treosulfan, of which two had either grade I or II acute GVHD. Further considerations to reduce graft failure have been proposed targeting the T cells where prominent activation may be observed, although controlling T cell overactivation prior to transplant would be desirable, it may not be realistic or feasible in clinical practice [26].

Ultimately, larger studies, evaluating both VEXAS with and without concurrent haematological malignancy are required along with detailed patient selection criteria. In the setting of an alternate rheumatological or autoimmune disease and transplantation for non-VEXAS cases, overall survival at 70%, and transplant-related mortality at 20.5% have been reported [27]. For patients with VEXAS alone and transplantation, this comprised of less than a third of patients in group 1, while remainder had concurrent haematological malignancy. Additionally, role of allo-HSCT patients with VEXAS and MDS requires further exploration, as this mode of treatment in lower-risk MDS are not favourable at 3 years, with overall survival at 58% and transplant-related mortality at 30%, respectively [28].

In the collected cases, the median time following transplant was relatively short, at only 10.9 months, with the longest follow-up in the prospective cases at 29.1 months. Therefore, while theoretically, transplantation may eliminate the *UBA1* mutation, further prospectively collected long-term data is needed. In terms of mortality, for group 1, there were three deaths (13.0%) with data available for one for which infection was implicated. It has also been reported that poor outcomes might also be observed due to chronicity of symptoms, in addition to advancing age and comorbidities, but also complications from years of immunosuppressive treatments [29].

A final remark pertains to the scarcity of data regarding post-HSCT monitoring in VEXAS. At least 6 patients revealed disappearance of *UBA1* mutations as early as 3–6 months after transplant, paralleling the reversion of the disease clinical phenotype [14]. Methods to track longitudinal clonal dynamics in VEXAS are not standardised and both Sanger (with the sensitivity caveat) or digital droplet PCR have been used [30]. As to chimerism, if a full chimerism is required even in cases without haematological malignancies is another unsolved matter as mixed chimerism has been reported to be linked with resolution of the disease, albeit with short follow-up thereby precluding the evaluation of whether the HSCT conditioning regimen may still be the reason of VEXAS phenotype reversion.

In summary, VEXAS syndrome is a complex, multifaceted disease entity often occurring with concurrent haematological malignancy. While allo-HSCT offers a promising treatment option, long-term data on acute and chronic complications, as well as guidelines around patient selection and personalised conditioning regimens are needed. A focused multidisciplinary approach is warranted to encompass the heterogeneity of VEXAS manifestations. Although the last 4 years have been a time of great proliferation of our understanding of VEXAS, there remains much to discover. Large, international collaborations with prospective data collection and rigorous clinical trials are required to delineate how best to diagnose and treat VEXAS despite its protean presentations.
